# Modeling structure and flexibility of *Candida antarctica *lipase B in organic solvents

**DOI:** 10.1186/1472-6807-8-9

**Published:** 2008-02-06

**Authors:** Peter Trodler, Jürgen Pleiss

**Affiliations:** 1 Institute of Technical Biochemistry, University of Stuttgart, Allmandring 31, D-70569 Stuttgart, Germany

## Abstract

**Background:**

The structure and flexibility of *Candida antarctica *lipase B in water and five different organic solvent models was investigated using multiple molecular dynamics simulations to describe the effect of solvents on structure and dynamics. Interactions of the solvents with the protein and the distribution of water molecules at the protein surface were examined.

**Results:**

The simulated structure was independent of the solvent, and had a low deviation from the crystal structure. However, the hydrophilic surface of CALB in non-polar solvents decreased by 10% in comparison to water, while the hydrophobic surface is slightly increased by 1%. There is a large influence on the flexibility depending on the dielectric constant of the solvent, with a high flexibility in water and a low flexibility in organic solvents. With decreasing dielectric constant, the number of surface bound water molecules significantly increased and a spanning water network with an increasing size was formed.

**Conclusion:**

The reduced flexibility of *Candida antarctica *lipase B in organic solvents is caused by a spanning water network resulting from less mobile and slowly exchanging water molecules at the protein-surface. The reduced flexibility of *Candida antarctica *lipase B in organic solvent is not only caused by the interactions between solvent-protein, but mainly by the formation of a spanning water network.

## Background

*Candida antarctica *lipase B (CALB) is an efficient catalyst for hydrolysis in water and esterification in organic solvents [1]. It is used in many industrial applications because of its high enantioselectivity, wide range of substrates, thermal stability, and stability in organic solvents [2]. CALB belongs to the α/β hydrolase fold family with a conserved catalytic triad consisting of Ser, His, and Asp/Glu [3]. The binding pocket for the substrates consists of an acyl binding pocket, a large and a medium binding pocket for the small and large moiety of secondary alcohols, respectively. In contrast to most lipases, CALB has no lid covering the entrance to the active site and shows no interfacial activation [4].

It has been shown that many enzymes retain activity in organic solvents [5] and have interesting catalytic properties such as higher thermostability and altered stereoselectivity [6,5,8], which was also observed for CALB [9,10]. As compared to water, organic solvents offer new possibilities in biocatalysis. In organic solvents, enzyme-catalyzed esterifications become feasible, and can be efficiently used due to the high solubility of hydrophobic substrates. Despite many advantages of enzymatic reactions in organic solvents, in most cases the catalytic activity in organic solvents is orders of magnitude lower than in aqueous systems [11-13], because of diffusional limitations, changes in protein flexibility, or destabilization of the enzyme [14,15]. It has been shown that the enzymatic activity in organic solvents can be correlated with the solvent polarity as indicated by the octanol-water partition coefficient logP [5]. However, it has also been reported that activity might be affected by the solvent without correlation to the logP [16,17]. Enzymes that are active in organic solvents retain their native structure upon transfer from water to organic solvents [18]. However, it is essential to add small amounts of water to maintain stability and flexibility of enzymes in organic solvent. Thus enzyme-bound water is essential for catalysis and serves as a lubricant for the enzyme [18]. In contrast, fully dry enzymes are inactive and enzymes in organic solvents with high amounts of water show denaturation [19].

Computer simulations of enzymes in different solvents are a valuable tool to investigate the effect on the structure and dynamics of proteins [20-23]. In most simulations of lipases, organic solvents are implicitly modeled [24]. In simulations with explicit organic solvent models, structural conservation and reduced flexibility in organic solvents as compared to water simulations have already been observed [25,24,31].

The aim of this work was to get insight in the structure and flexibility of CALB in water and five different organic solvents using multiple molecular dynamics simulations. CALB was selected in this study, because of its industrial relevance and its stability in different organic solvents. Using explicit solvent models and a low amount of protein-bound water molecules allows to analyze the position of single water molecules, their exchange during the simulation, and the effect of molecular properties of the organic solvents.

## Results

### Structure of CALB

**Figure 1:**
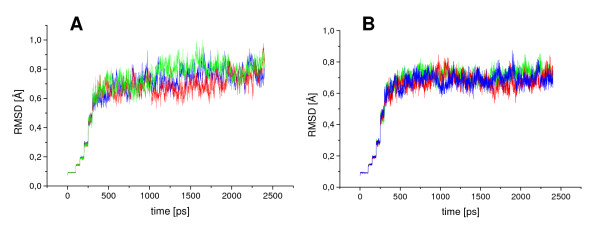
**RMS-Deviation in simulations of CALB**. Root mean squared deviations of CALB backbone atoms from the X-ray structure as a function of time during simulations of 2 ns in (A) water and (B) in toluene showing three different simulations of the same system using different initial velocity distributions, indicated by different colors

**Figure 2:**
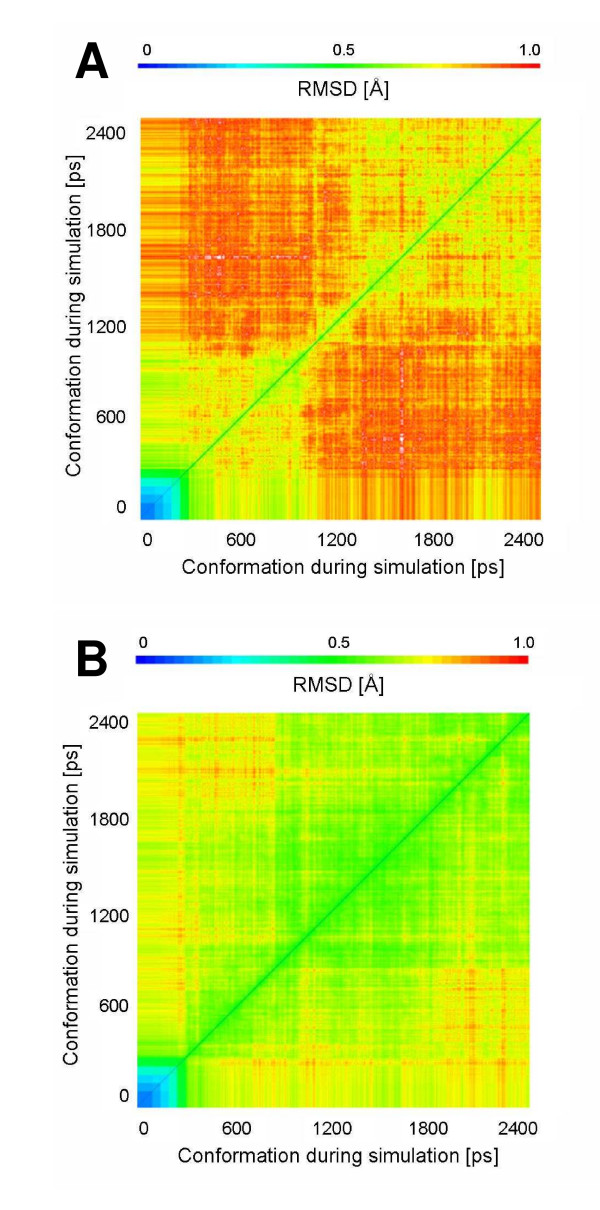
**2D-RMSD in simulations of CALB**. The root mean square deviation of every conformation to all other conformations of a simulation, as a function of time of CALB backbone atoms during a 2 ns simulation in (A) water and (B) cyclohexane, is shown in the 2D-RMSD

In the simulations of CALB in water (WAT) (Figure 1) and in the polar solvent methanol (MET), the root mean square deviation (RMSD) of the backbone atoms to the initial structure increased gradually during 1 ns. After this equilibration phase the RMSD in WAT and MET were in the range from 0.7 to 0.9 Å and CALB was in equilibrium without showing any significant conformational changes. The simulations of CALB in the non-polar solvents chloroform (CL3), isopentane (ISO), toluene (TOL), and cyclohexane (CHE) reached the equilibrium already after 500 ps (Figure 1). After equilibration, the RMSD to the initial structure were in the range from 0.7 to 0.9 Å, which is slightly smaller than simulations in WAT and MET. No significant structural differences of CALB backbone in all solvents were observed, and the RMSD between the conformations of CALB in all solvents were in a range from 0.4 to 0.8 Å. The 2D-RMSD plot, where the root mean square deviation of every conformation to all other conformations of a simulation is shown, demonstrated that the conformational space sampled by CALB in the simulations in WAT and MET was larger than in non-polar solvents (Figure 2). In CALB, hydrophobic and hydrophilic patches are equally distributed over the surface (Additional file 1) and the solvent accessible surface area of hydrophilic and hydrophobic surface residues is similar (Table 1). In the simulations in WAT the total surface increased by 600 Å^2 ^compared to the crystal structure, while in non-polar solvents the total surface is similar to the crystal structure. After simulations in non-polar organic solvents the hydrophilic surface is decreased by about 600 Å^2 ^as compared to simulations in WAT.

**Table 1:** Total and hydrophilic surface of CALB in the crystal structure and averaged over the last 1 ns of each simulation in six solvents. Hydrophilic residues are by negative Eisenberg

solvent	total surface [Å^2^]	hydrophilic surface [Å^2^]
crystal structure	12043	6071
water	12659	6410
methanol	12564	6069
chloroform	12281	5828
isopentane	12157	5858
toluene	12115	5838
cyclohexane	12014	5710

### Flexibility of CALB

**Figure 3:**
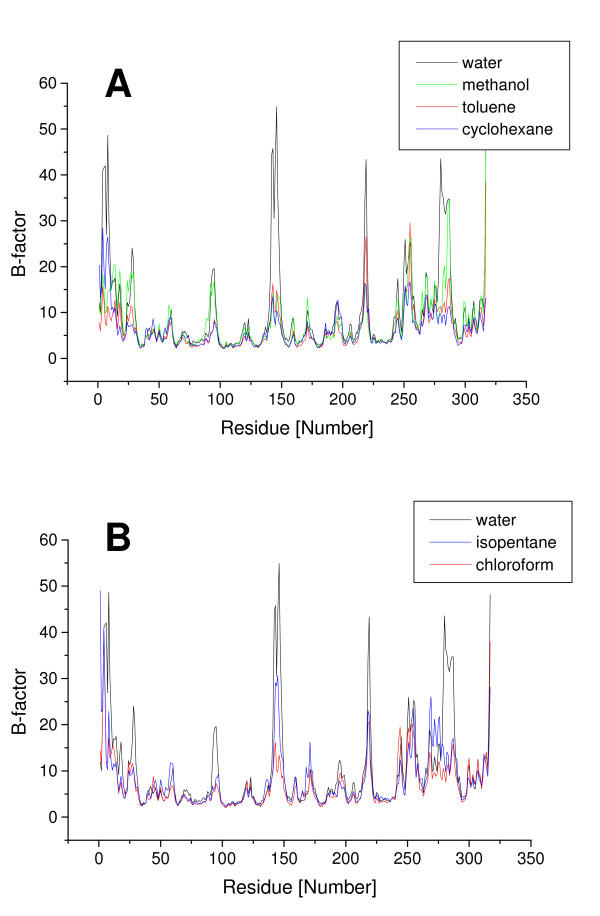
**Flexibility of CALB in different solvents**. Flexibility (indicated by B-factors per residue) of CALB calculated from the last 1 ns of simulations (A) in water (black), in the polar solvent methanol (green), and in the non-polar organic solvents toluene (red) and cyclohexane (blue) and (B) the non-polar organic solvents chloroform (red) and isopentane (blue)

**Figure 4:**
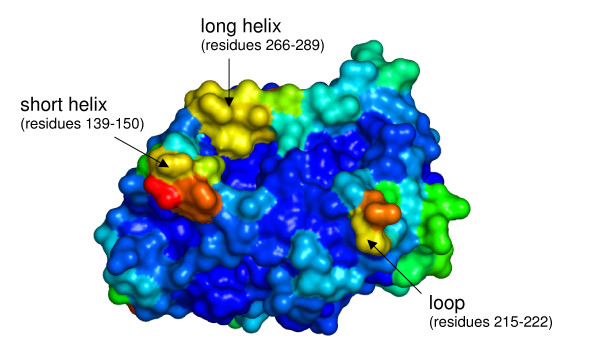
**Flexible regions in CALB**. Flexibility (indicated by B-factors per residue) of CALB from simulations in water mapped on the surface of the crystal structure (blue: low, red: high flexibility); highly flexible including regions are labeled

To evaluate the flexibility of CALB in different solvents, B-factors per residue were calculated from the simulations. In all solvents CALB showed lower flexibility in the core and the active site, and higher flexibility of residues located on the surface. In the WAT, the overall flexibility, as estimated from the sum of all B-factors, is significantly higher than in the organic solvents (Table 2). The overall flexibility of CALB decreases in organic solvents in the order methanol, isopentane, chloroform, toluene, and cyclohexane. The regions with higher flexibility are the same in every solvent, but the degree of decreased flexibility is different. While the flexibility of the rigid core residues is similar in all solvents, the solvent-dependent changes of flexibility are limited to only 5 surface elements (Figure 3): a short α-helix (residues 139–150), a long α-helix (residues 266–289) which form the entrance to the active site and three surface loops (residues 26–30, and 92–97, 215–222), showing higher difference in flexibility between water and non-polar solvents. The medium binding pocket for secondary alcohols (residues 39, 42, 47, 104, 225) and the acyl binding pocket (residues 134, 138, 157, 189, 190) showed low flexibility, while the large binding pocket for secondary alcohols (residues 141,144,154,285,289,290) showed higher flexibility (Figure 4).

**Table 2:** Total B-factors (sum of B-factors per residue) of CALB averaged over the last 1 ns of each simulation in six solvents

CALB in solvent	total B-factor [Å^2^]
water	5267 ± 396
methanol	4385 ± 122
isopentane	4013 ± 78
chloroform	3648 ± 215
toluene	3318 ± 103
cyclohexane	3216 ± 80

**Figure 5:**
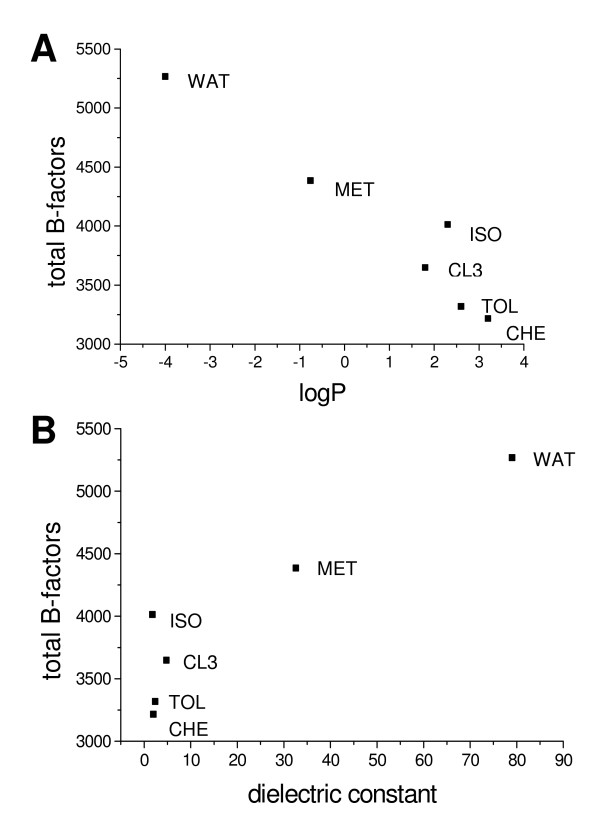
**Dependence of CALB flexibility on logP and dielectric constant**. Flexibility of CALB (sum of B-factors) calculated from the last 1 ns of simulations as a function of (A) logP and (B) dielectric constant from simulations in different solvents. logP values and dielectric constants were taken from the CRC Handbook of Chemistry and Physics [75]

The flexibility in different solvents was correlated to logP and the dielectric constant of the solvents. The simulations showed a decrease of CALB flexibility by increasing logP and a decreasing dielectric constant (Figure 5). The simulations in ISO are an exception, which will be discussed later. For all twenty amino acids, the highest flexibility was observed in the WAT. However, the extent of the decrease of flexibility in non-polar organic solvents depends on the type of amino acid. While in non-polar solvents the flexibility of glutamine and the hydrophobic amino acids V, Y, T, F, L, I, and W decreased only slightly to 80, 73, 74, 76, 80, 81, 82 and 98%, respectively, of their flexibility in water, the flexibility of alanine and the hydrophilic amino acids D, R, E and N was significantly decreased in non-polar organic solvents to 54, 44, 55, 56 and 62%.

### Water at the surface

**Figure 6:**
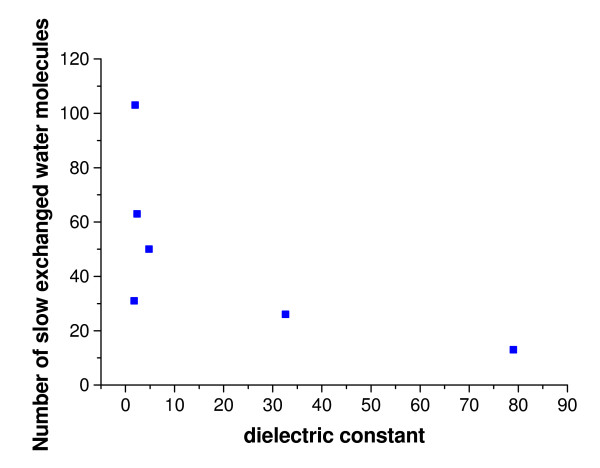
**Dependence of slow exchanged water molecules on dielectric constant**. Number of less mobile water molecules at the surface of CALB, with a B-factor lower than 40 Å^2 ^calculated from the last 1 ns of simulations, as a function of the dielectric constant from simulations in different solvents

**Figure 7:**
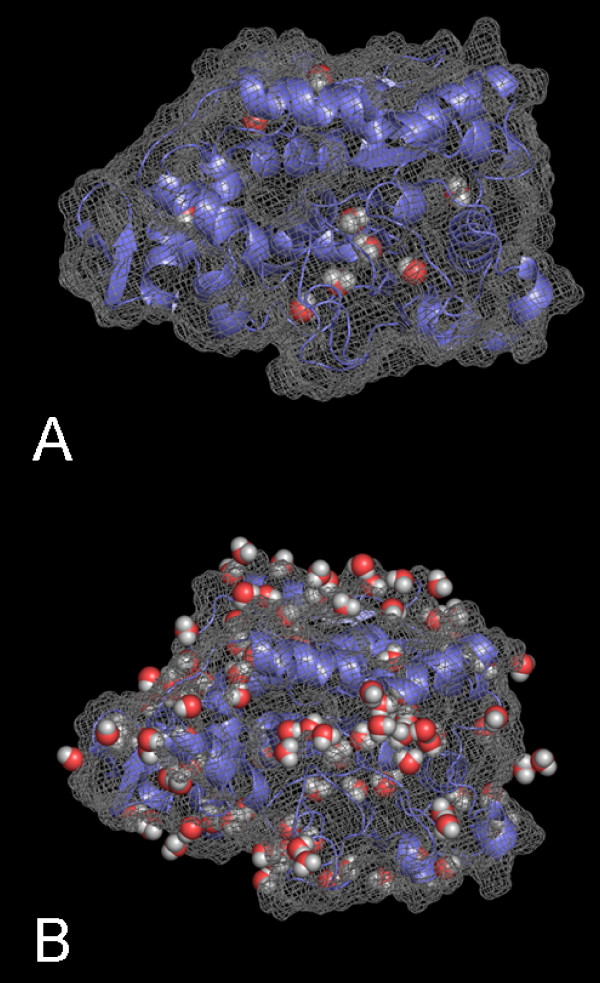
**Less mobile water molecules at the surface of CALB**. Location of less mobile water molecules, with a B-factor lower than 40 Å^2^, at the surface of the last snapshot of CALB during the simulation (A) in water and (B) in cyclohexane; water molecules are displayed as red and white spheres

**Figure 8:**
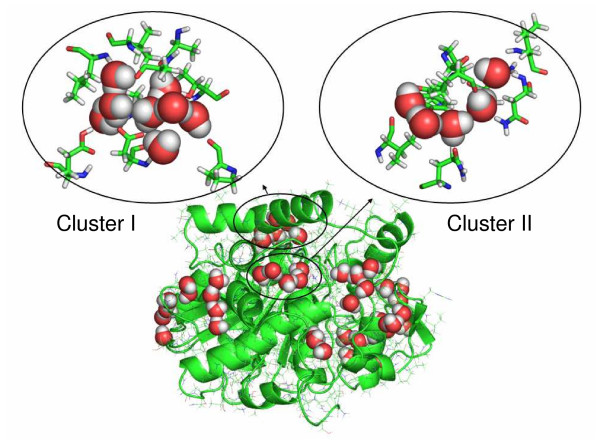
**Water network and water clusters on CALB**. Snapshot of the spanning water network during the simulation of CALB in cyclohexane with representative clusters I and II, which consist of surface-bound and bridging water molecules; water molecules are displayed as red and white spheres

During the simulations in WAT and in organic solvents, the nine buried water molecules which have been identified in the crystal structure [3] were not exchanged. In contrast, all water molecules that were initially bound at the surface were exchanged by other water molecules during the simulations in the WAT, either by water molecules from the bulk or by other surface-bound water molecules. In non-polar solvents the water exchange at the surface is less frequent and the bound water molecules were less mobile depending on the type of solvent (Figure 6), using a B-factor of 40 Å^2 ^as cutoff. While in WAT there were only 13 ± 2 bound water molecules (averaged over three simulations) with a low B-factor less than 40 Å^2 ^identified, this number increased with increasing logP of the solvent to 26 ± 2 in MET, 31 ± 7 in ISO, 50 ± 17 in CL3, 63 ± 9 in TOL, and 103 ± 6 in CHE out of a total of 286 water molecules included in the organic solvent simulations. However, while the number of less mobile water molecules at the protein surface was similar in the three simulations of the same system, the binding sites of these water-molecules were different. In CHE simulations the 103 less mobile surface water molecules formed a spanning water network covering the whole surface of CALB (Figure 7). This spanning water network consists of water molecules bound to polar side chains or to the backbone of the enzyme, as well as of water molecules bridging these bound waters (Figure 8). The spanning water network is variable and differs slightly in each simulation of the same protein-solvent system. Due to the lower number of less mobile water molecules at the CALB surface in TOL, ISO, and CL3, the spanning water network decreased in size with an decreasing logP. A spanning water network was not observed in MET and WAT simulations. Water clusters include water molecules bound to the protein and water bridges between two water molecules. In CHE eight water clusters of less mobile water molecules were identified by visual inspection (Figure 8). The largest cluster consisted of eight water molecules, where five water molecules were directly bound to polar side chains or to backbone atoms, while three water molecules are bridging these protein-bound waters, but did not interact with the protein itself (Additional file 2, 3, 4, 5). Visual inspection indicated that the active site is accessible to organic solvents. In simulations in MET five methanol and two water molecules were located in the substrate binding site after 2 ns of simulation, in CL3 one chloroform and five water molecules, in ISO one isopentane and six water molecules, in TOL one toluene and five water molecules, and in CHE one cyclohexane and six water molecules. In MET four water molecules that were initially present in the substrate binding site were replaced by methanol, which was not observed by non-polar solvent molecules.

## Discussion

Three simulations with different initial velocity distributions instead of a single trajectory were used to sample the conformational space in the protein-solvent system, because a single trajectory samples only a small fraction of the conformational space than multiple short trajectories [32-34].

The structure of CALB shows a high stability in all solvents and is therefore a useful system to examine the effect of different solvents on structure and flexibility. Multiple MD simulations of each protein-solvent system confirmed that the structures of CALB in different solvent deviated from each other by less than 0.8 Å. This structural difference is within the deviation of 0.6–0.8 Å of the three simulations of the same protein-solvent system. Our observation that the structure of CALB is independent of its environment is supported by the fact that CALB does not undergo conformational transitions [4] and by a comparison of different crystal structures obtained under different crystallization conditions [3,35] which show backbone RMSD values below 0.3 Å. Also in most molecular dynamics simulations structures showed no significant changes in different solvents [20,22,31] with the exception of *Rhizomucor miehei *lipase for which a solvent-induced conformational change was observed [30]. In addition, circular dichroism measurements of CALB in different solvents showed that its secondary structure did not change [36]. This is also confirmed by X-ray structures of various serine proteases, crystallized in the presence of small amounts of different organic solvents, which are nearly indistinguishable from their structure in water [37-40]. Flexible and rigid regions of CALB identified in the simulations were similar to regions of high and low B-factors reported in the crystal structure [3]. In contrast to structure, the flexibility is solvent-dependent. In organic solvents, the flexibility of proteins is decreased, which has been confirmed by different experimental techniques such as time-resolved fluorescence anisotropy [41], ESR [42], and dielectric relaxation spectroscopy [43]. This was also observed in simulations of lipases [30] and subtilisin [20], while no significant differences between water and organic solvents have been observed in simulations of subtilisin [44].

It is observed that solvents with a lower dielectric constant lead to a decreased protein flexibility as shown by EPR [45,12], which is in general consistent with the results of our simulations. However, there is one outlier. In ISO the flexibility is higher than expected from its dielectric constant. Interestingly, isopentane is the only solvent in our simulations with one freely rotatable, bond which is not considered in the rigid solvent model. We suppose that a flexible solvent model would be necessary to properly treat the effects of this solvent. It has also been suggested that the size of organic solvent molecules correlates with the protein flexibility [46], but no correlation was found in our simulations. A further effect of organic solvents in our simulations is a decrease in solvent-accessible surface, especially of the hydrophilic surface. In our simulations, the hydrophilic surface decreased by 600 Å^2 ^from simulations in WAT to CHE, while the hydrophobic surface increased slightly by 50 Å^2^. It has been suggested that in water polar side chains orient toward the surface, thus increasing the hydrophilic surface and decreasing the polar intra-molecular interactions that mediate the rigidity of the protein [47], while in organic solvents the surface area is reduced which leads to improved packing and increased stability [42].

There are in general two types of water molecules observed in organic solvents. The 'inside class' water molecules that are bound in the interior of the protein and can play an important role for active conformation in organic solvents by a hydrogen bond network and the 'contact class' water molecules that are weakly bound to the surface of the enzyme and can be rapidly exchanged by other water molecules [48]. The dynamical properties of surface-bound water molecules differ considerably from the bulk water as shown by X-ray crystallography [49] and NMR experiments [50]. The residence times of most surface-bound water molecules are between 10 and 100 ps, bound and free water molecules at the surface of a protein are in a dynamic equilibrium [51]. In agreement with these experiments all water molecules at the surface were rapidly exchanged during the simulation time of 2 ns. In correlation to our simulations, where an increased number of less mobile water molecules was observed at higher logP at the surface of CALB, the number of water molecules with high B-factor was decreased by an increasing hydrophobicity of alcohols at lysozyme studied by X-ray [52]. In organic solvents the amount of water molecules in the organic solvent phase is low and therefore the probability for an exchange of bound and structured water at the surface is low. A high rate of exchange of water molecules at the surface, observed in our simulations, might be the reason for an increased flexibility, in agreement to previous observations where the protein mobility increased with an increasing amount of water [22].

In agreement with experimental results [14] in the simulations organic solvents strip just a few water molecules from the enzymes surface, while polar solvents strip most water from the surface. This stripping of water from the surface by polar solvents has been already shown in experiments [12] and in simulations [31]. The solvent dielectric constant correlated with stripping of water molecules in simulations with increasing polarity of the solvent [44]. It was shown in experiments, that desorption is independent of the kind of the protein, increasing with the dielectric constant of the solvent [53]. Like in our simulations of CALB, a spanning water network consisting of several small water clusters was observed in previous simulations [54,55,22,31] and experiments [49], in which non-polar solvents enhanced the formation of clusters [31,54]. In agreement to our simulations, the water clusters in a cutinase consisted of 2 to 8 water molecules at a hydration level of 15% (w/w) [31], depending on the solvent, in the crystal structure of CALB the largest cluster of water molecules with B-factors lower 40 Å^2 ^consists of 14 water molecules at most. The spanning water network resulted by a slow exchange of water molecules at the surface in organic solvents. Polar groups favor direct interactions with water molecules and form hydrogen bonds, while non-polar groups enhance interactions among water molecules and enhance the local structure of neighboring water molecules. The concept of hydrophobic hydration and the freezing of water to clusters around hydrophobic surface was previously suggested [56] and is supported by the solvent dependent flexibility observed in simulations.

From the results it can be concluded, that the reduced flexibility of CALB in non-polar solvents is not only a consequence of the interaction between organic solvent molecules and the protein, but also due to the interaction with the enzyme-bound water and its exchange on the surface. Despite the higher fluidity of organic solvents, the flexibility of CALB is decreased, because the water exchange at the surface is restricted.

## Methods

### Parametrization of solvent models

The organic solvent molecules of cyclohexane, isopentane, and toluene were parameterized. The geometric parameters were derived by *ab initio *geometry optimization on the HF/6-31G* level using Gaussian98 [57] in the gas phase. The partial charges were derived by fitting partial charges using the RESP program [58] of AMBER 7.0 [59] to the electrostatic potential (Additional file 6). For each molecule a periodic solvent box was built in XLEAP (Additional file 7). The boxes were equilibrated by molecular dynamics simulations using AMBER 7.0 and the all-atom AMBER force-field ff99 [60] by pressure coupling, all solvent molecules were treated as rigid. The calculated densities of the boxes in equilibrium were in good agreement with experimental data (Additional file 7). After minimization (500 steps steepest descent followed by 200 steps conjugate gradient) the systems were heated during 30 ps to 300 K using a temperature coupling constant of 0.8 ps at a pressure of 1 bar using a pressure coupling constant of 1.0 ps [61]. Molecular dynamics simulations of the systems were performed for 2 ns, applying the SHAKE algorithm [62] to constrain the bond lengths. Electrostatic interactions were calculated using Ewald summation [59], Van der Waals interactions were calculated using a 16 Å cut-off.

### System setup

The crystal structure of CALB [PDB: 1TCA] [3] with a resolution of 1.55 Å was taken from the Protein Data Bank as initial structure for the simulations.

pKa values and protonation states of titratable groups Arg, Lys, Asp, Glu, and His were calculated at pH 7 using MEAD [63] and TITRA [64]. The online tool PCE [65] of MEAD was used to solve numerically the Poisson-Boltzmann equation using its MULTIFLEX program with a protein internal dielectric constant ε = 20, solvent dielectric constant ε = 80, ionic strength 0.145 M, grid spacing of 1 Å in a cubic box and the PARSE parameters for radii and charges [66]. The REDTI program was used to compute the protonation states [67]. TITRA is based on the Tanford-Kirkwood model, with default parameters and ε = 20 for the protein dielectric constant. The solvent accessible surface area of each residue used in TITRA was calculated by the program acc_run [68]. Both methods resulted in the same protonation states at pH 7. CALB was calculated to be neutral, the same protonation states were used for the simulations in water and in organic solvents, assuming pH memory from the protonation in aqueous solution after lyophilization [14]. In XLEAP of the Amber 7.0 program package hydrogens were added as calculated by TITRA. The CALB crystal structure including 286 crystal water molecules, corresponding to a hydration level of 15.4% (w/w), was solvated in six different solvent boxes using a minimal distance of 14 Å between the box boundary and the protein. The equilibrated boxes of cyclohexane (CHE), isopentane (ISO), and toluene (TOL) were used as parametrized, boxes of TIP3 water (WAT) [69], methanol (MET) [70] and chloroform (CL3) [71] were used as given in the AMBER package.

### Molecular dynamics simulations

Multiple molecular dynamics simulations of the protein-solvent systems were performed using the Amber 7 program package [59] and the all-atom AMBER force field ff99 [60]. The simulations were done in a truncated octahedral box under periodic boundary conditions. Non-bonded interactions were calculated at a cutoff distance of 10 Å. The SHAKE algorithm [62] was applied to all bonds. The simulations were performed at 300 K and 1 bar using a time step of 1 fs. Temperature and pressure of the system were controlled using a weak coupling to an external heat bath [61] with a temperature coupling constant of 1.0 ps and a pressure coupling constant of 1.2 ps. The initial structures were energy minimized (500 steepest descent and 50 conjugate gradient) and followed by a simulation at 300 K and 1 bar by restraining the position of all C_α _atoms using a harmonic potential. The force constant was gradually decreased every 50 ps from 10 to 5, 1 and 0.1 kcal/mol followed by an unrestrained simulation of 2 ns. Three simulations of each system were performed using different initial random velocity distributions. All snapshots from the resulting trajectories were fitted the backbone atoms to the initial structure. The root mean square deviation of the backbone atoms between each conformer and the initial structure (RMSD) and between all conformers (2D-RMSD) and B-factors were analyzed using the PTRAJ of AMBER 7.0 [59]. The solvent accessible surface area was calculated by DSSP [72] using a probe radius of 1.4 Å. The protein structures were visualized and the hydrophobicity [73] was mapped on the surface using PyMol 0.98 [74]. Hydrophilic and hydrophobic residues were identified by their negative or positive hydrophobicity index [73], respectively.

## Authors' contributions

PT performed the simulations, JP was the principal investigator, conceived the project and guided its development. All authors read and approved the final manuscript.

## Supplementary Material

Additional file 1 Hydrophobicity of CALB. Surface of CALB colored by hydrophobicity (hydrophobic parts red; hydrophilic parts white) Click here for file

Additional file 2 Cluster I – Structure. Structure of cluster I in the simulation of CALB in cyclohexane, water molecules are displayed as red and white spheres Click here for file

Additional file 3 Cluster I – Ligands. Coordination of water molecules in cluster I in the simulation of CALB in cyclohexane Click here for file

Additional file 4 Cluster II – Structure. Structure of cluster II in the simulation of CALB in cyclohexane, water molecules are displayed as red and white spheres Click here for file

Additional file 5 Cluster II – Ligands. Coordination of water molecules in cluster II in the simulation of CALB in cyclohexane Click here for file

Additional file 6 Parameters of solvent models. The parameters of the solvent models cyclohexane, isopentane and toluene parametrized by *ab initio *geometry optimization Click here for file

Additional file 7 Data of solvent Boxes. Equilibration of solvent boxes by molecular dynamics simulations Click here for file
