# Supplementary material for: Modeling structure and flexibility of Candida antarctica lipase B in organic solvents
Source: BMC Struct Biol. 2008 Feb 6;8:9. doi: 10.1186/1472-6807-8-9 (PMC2262892; doi:10.1186/1472-6807-8-9)
Supplement: Additional file 1 — Hydrophobicity of CALB. Surface of CALB colored by hydrophobicity (hydrophobic parts red; hydrophilic parts white) [file 1472-6807-8-9-S1.pdf]

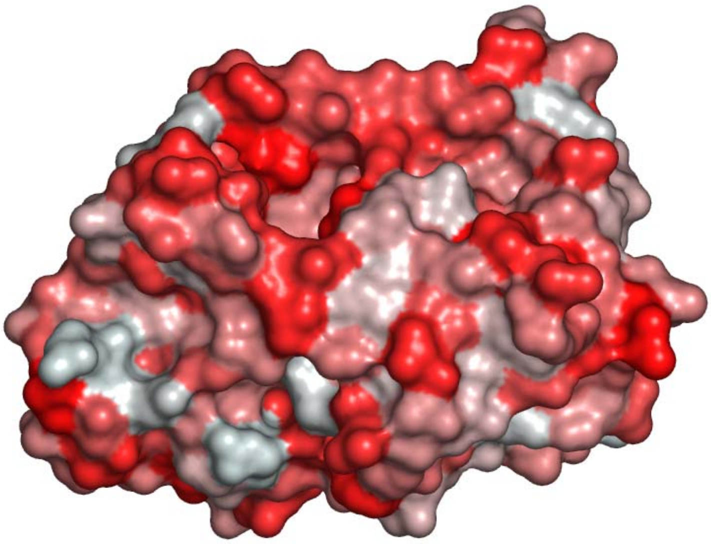

Additional file 1
Surface of CALB colored by hydrophobicity (hydrophobic, red; hydrophilic, white)
