# Supplementary material for: Modeling structure and flexibility of Candida antarctica lipase B in organic solvents
Source: BMC Struct Biol. 2008 Feb 6;8:9. doi: 10.1186/1472-6807-8-9 (PMC2262892; doi:10.1186/1472-6807-8-9)
Supplement: Additional file 2 — Cluster I – Structure. Structure of cluster I in the simulation of CALB in cyclohexane, water molecules are displayed as red and white spheres [file 1472-6807-8-9-S2.pdf]

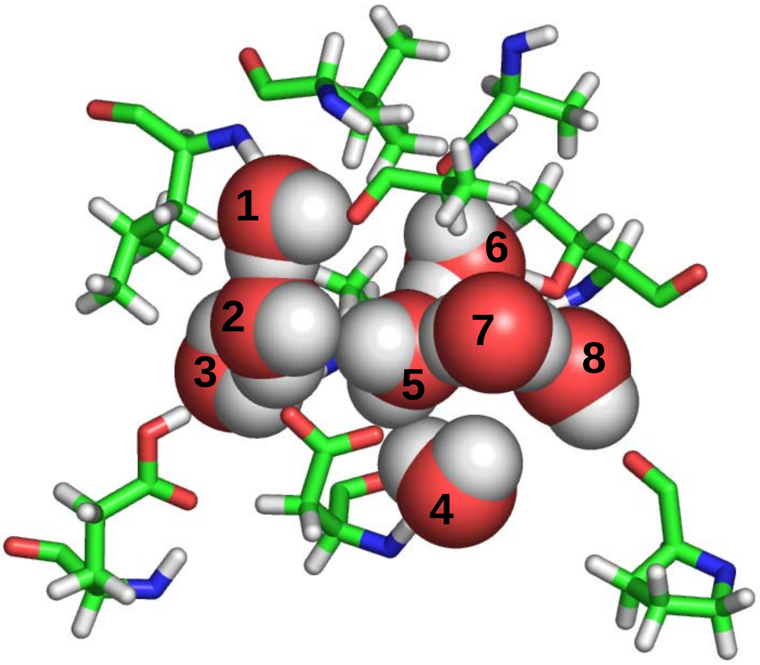

Additional file 2

Structure of cluster I in the simulation of CALB in cyclohexane, water molecules are displayed as red and white spheres
