# Supplementary material for: Modeling structure and flexibility of Candida antarctica lipase B in organic solvents
Source: BMC Struct Biol. 2008 Feb 6;8:9. doi: 10.1186/1472-6807-8-9 (PMC2262892; doi:10.1186/1472-6807-8-9)
Supplement: Additional file 3 — Cluster I – Ligands. Coordination of water molecules in cluster I in the simulation of CALB in cyclohexane [file 1472-6807-8-9-S3.pdf]

**Additional file 3**

Coordination of water molecules in cluster I

| water molecule | ligand 1 | ligand 2 | ligand 3 | ligand 4 |
|---|---|---|---|---|
| 1 | Glu188 side chain | Asp223 side chain | Ala225 backbone | water molecule 2 |
| 2 | | water molecule 1 | water molecule 3 | water molecule 4 |
| 3 | Leu278 backbone | Leu277 backbone | Ala275 backbone | water molecule 2 |
| 4 | water molecule 3 | water molecule 5 | water molecule 7 | water molecule 8 |
| 5 | Thr229 side chain | Ala274 backbone | water molecule 4 | water molecule 6 |
| 6 | Pro260 backbone | water molecule 5 | water molecule 7 | |
| 7 | water molecule 4 | water molecule 5 | water molecule 6 | water molecule 8 |
| 8 | Asp223 backbone | Asp223 side chain | water molecule 6 | water molecule 7 |
