# Supplementary material for: Modeling structure and flexibility of Candida antarctica lipase B in organic solvents
Source: BMC Struct Biol. 2008 Feb 6;8:9. doi: 10.1186/1472-6807-8-9 (PMC2262892; doi:10.1186/1472-6807-8-9)
Supplement: Additional file 4 — Cluster II – Structure. Structure of cluster II in the simulation of CALB in cyclohexane, water molecules are displayed as red and white spheres [file 1472-6807-8-9-S4.pdf]

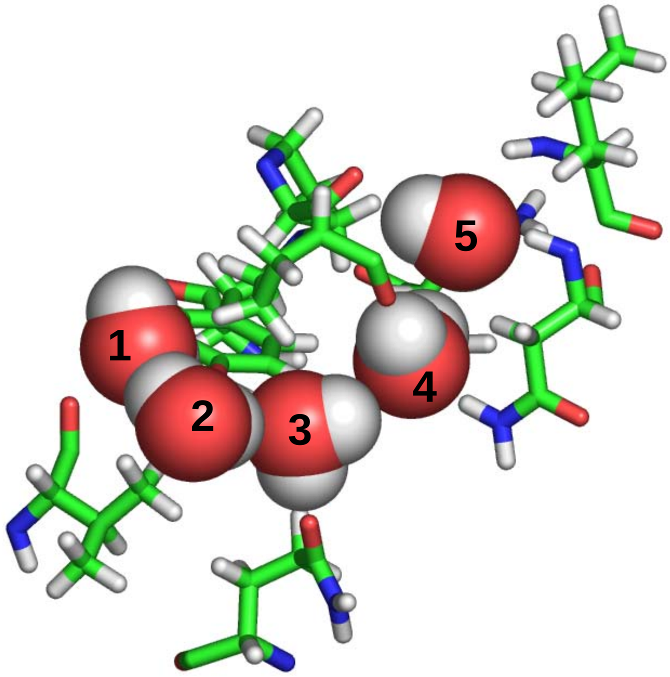

Additional file 4

Structure of cluster II in the simulation of CALB in cyclohexane, water molecules are displayed as red and white spheres
