# Supplementary material for: Modeling structure and flexibility of Candida antarctica lipase B in organic solvents
Source: BMC Struct Biol. 2008 Feb 6;8:9. doi: 10.1186/1472-6807-8-9 (PMC2262892; doi:10.1186/1472-6807-8-9)
Supplement: Additional file 5 — Cluster II – Ligands. Coordination of water molecules in cluster II in the simulation of CALB in cyclohexane [file 1472-6807-8-9-S5.pdf]

**Additional file 5**

Coordination of water molecules in cluster II

| water molecule | ligand 1 | ligand 2 | ligand 3 | ligand 4 |
|---|---|---|---|---|
| 1 | Val15 backbone | Tyr82 side chain | water molecule 2 | |
| 2 | water molecule 1 | water molecule 3 | | |
| 3 | Gln11 side chain | Tyr82 side chain | water molecule 2 | water molecule 4 |
| 4 | Asn74 Seitenkette | Pro69 backbone | water molecule 3 | water molecule 5 |
| 5 | Pro68 backbone | Asn74 backbone | Asn74 side chain | water molecule 4 |
