# Supplementary material for: Modeling structure and flexibility of Candida antarctica lipase B in organic solvents
Source: BMC Struct Biol. 2008 Feb 6;8:9. doi: 10.1186/1472-6807-8-9 (PMC2262892; doi:10.1186/1472-6807-8-9)
Supplement: Additional file 6 — Parameters of solvent models. The parameters of the solvent models cyclohexane, isopentane and toluene parametrized by ab initio geometry optimization [file 1472-6807-8-9-S6.pdf]

Additional file 6

Parameters of solvent models

A) **Cyclohexane**

Atom names in cyclohexane:

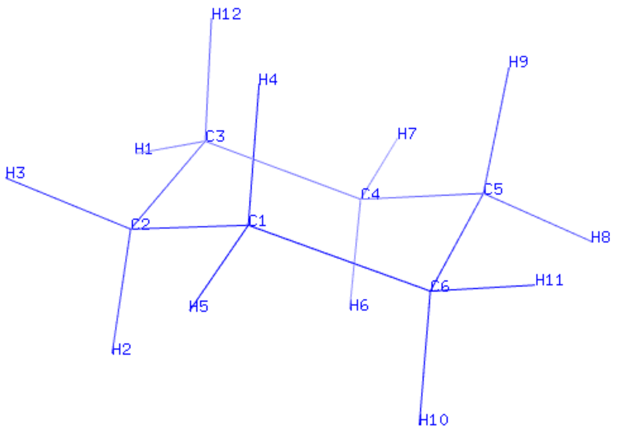

Atom name	AMBER atom type	Partial charge
C1	CT	0.02395
H4	HC	-0.01198
H5	HC	-0.01198
C2	CT	0.02395
H2	HC	-0.01198
H3	HC	-0.01198
C3	CT	0.02395
H1	HC	-0.01198
H12	HC	-0.01198
C4	CT	0.02395
H6	HC	-0.01198
H7	HC	-0.01198
C5	CT	0.02395
H8	HC	-0.01198
H9	HC	-0.01198
C6	CT	0.02395
H10	HC	-0.01198
H11	HC	-0.01198

Bond	Bond length [Å]
C-C	1,53
C-H	1,09

B) **Isopentane**

Atom names in isopentane:

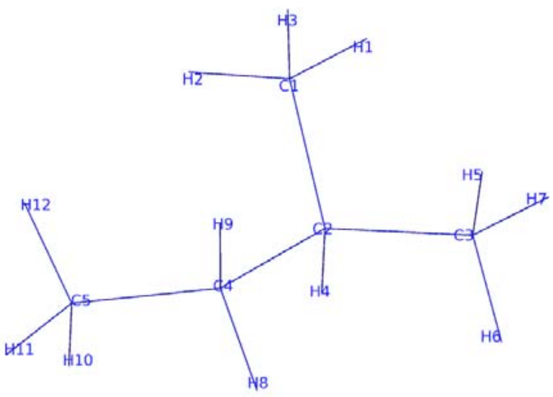

Atom name	AMBER atom type	Partial charge
C1	CT	-0.447
H1	HC	0.102
H2	HC	0.102
H3	HC	0.102
C2	CT	0.380
H4	HC	-0.038
C3	CT	-0.447
H5	HC	0.102
H6	HC	0.102
H7	HC	0.102
C4	CT	-0.068
H8	HC	0.016
H9	HC	0.016
C5	CT	-0.102
H10	HC	0.026
H11	HC	0.026
H12	HC	0.026

Bond	Bond length [Å]
C-C	1,53
C-H	1,09

C) Toluene

Atom names in toluene

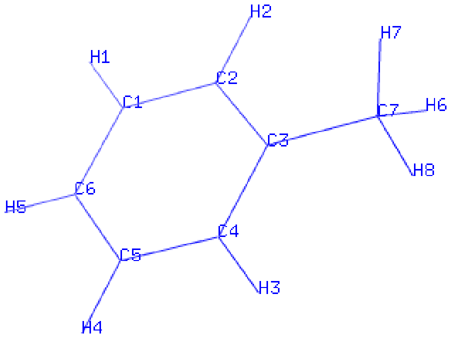

Atom name	AMBER atom type	Partial charge
C2	CA	-0.27587
H2	HA	0.15626
C1	CA	-0.11100
H1	HA	0.13753
C6	CA	-0.17752
H5	HA	0.14095
C5	CA	-0.11100
H4	HA	0.13753
C4	CA	-0.27587
H3	HA	0.15626
C3	CA	0.28974
C7	CT	-0.38897
H6	HC	0.10732
H7	HC	0.10732
H8	HC	0.10732

Bond	Bond length [Å]
C3-C7	1,51
C7-H	1,09
C3-C2	1,39
C2-H2	1,08
C2-C1	1,39
C1-H1	1,08
C1-C6	1,38
C6-H6	1,08
