# Supplementary material for: Modeling structure and flexibility of Candida antarctica lipase B in organic solvents
Source: BMC Struct Biol. 2008 Feb 6;8:9. doi: 10.1186/1472-6807-8-9 (PMC2262892; doi:10.1186/1472-6807-8-9)
Supplement: Additional file 7 — Data of solvent Boxes. Equilibration of solvent boxes by molecular dynamics simulations [file 1472-6807-8-9-S7.pdf]

Additional file 7

Equilibration of solvent boxes by molecular dynamics simulations

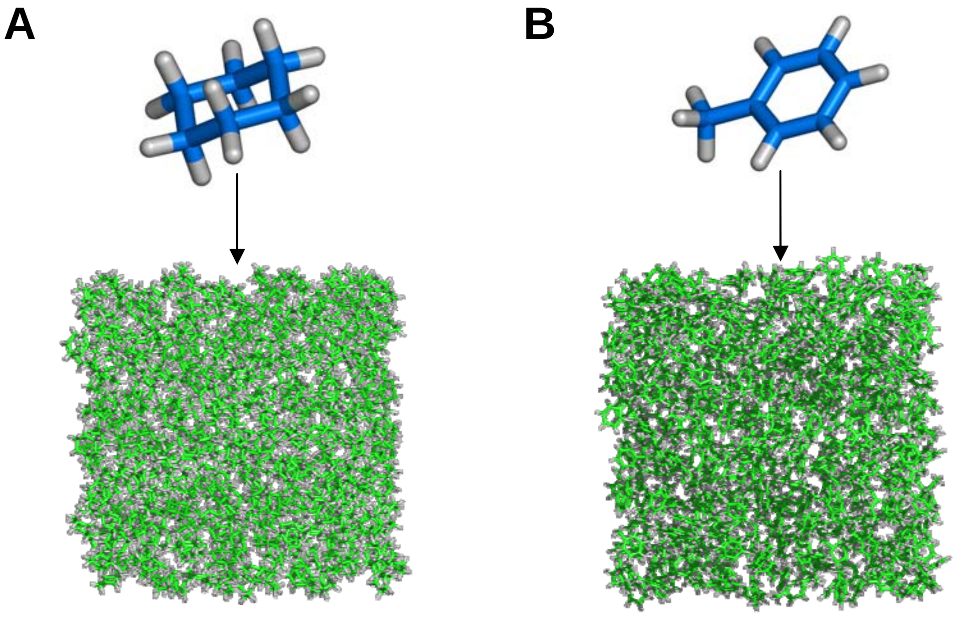

Figure Solvent molecules (blue) and equilibrated solvent boxes by molecular dynamics simulations (green) of cyclohexane (A) and toluene (B)

Table: Number of solvent molecules in simulated solvent boxes

Solvent	Number of solvent molecules in the box	Edge length of the box after simulation [Å]
Cyclohexane	379	41.1
Isopentane	351	40.7
Toluene	436	46.4

Table: Comparison of the experimental density and the density of parametrized solvent models after simulation. Experimental densities were taken from the product properties at www.sigmaaldrich.com.

Solvent	Density after simulation [g/cm^3]	Experimental density [g/cm^3]
Cyclohexane	0.76	0.78
Isopentane	0.63	0.63
Toluene	0.84	0.86
